# Value of Sarcopenia defined by the new EWGSOP2 consensus for the prediction of Postoperative Complications and Long-term Survival after Radical Gastrectomy for Gastric Cancer: A comparison with four common nutritional screening tools

**DOI:** 10.7150/jca.49815

**Published:** 2020-08-06

**Authors:** Dong-Dong Huang, Hui-Yang Cai, Xi-Yi Chen, Wen-Xi Dong, Drugyel Wangchuk, Jing-Yi Yan, Xiao-Lei Chen, Qian-Tong Dong

**Affiliations:** 1 Department of Gastrointestinal Surgery, The First Affiliated Hospital of Wenzhou Medical University, Wenzhou, China.; 2 Department of Cardiothoracic Surgery, The Second Affiliated Hospital of Wenzhou Medical University, Wenzhou, China.

**Keywords:** Sarcopenia, Malnutrition, Gastrectomy, Postoperative complication, Survival

## Abstract

**Background:** Nutritional risk and sarcopenia are both associated with increased postoperative morbidity and mortality following elective surgery. This study aimed to investigate whether sarcopenia has additional predictive value for postoperative complications and long-term survival besides nutritional screening tools.

**Methods:** Clinical data of patients underwent radical gastrectomy for gastric cancer was prospectively collected. Sarcopenia was diagnosed by grip strength plus muscle quanlity/quality based on preoperative abdominal CT scans. Nutritional screening was performed using 4 common nutritional screening tools, including Malnutrition Universal Screening Tool (MUST), Nutritional Risk Screening (NRS)-2002, Malnutrition Screening Tool (MST), and Short Nutritional Assessment Questionnaire (SNAQ).

**Results:** A total of 880 patients were analyzed, in which 167 (18.98%) were diagnosed with sarcopenia. The incidence of nutritional risk identified by the 4 tools were 44.66% (MUST ≥1), 35.23% (NRS-2002 ≥3), 29.89% (MST ≥2), and 20.34% (SNAQ ≥2). Multivariate analyses showed that nutritional risk identified by the 4 nutritional screening tools were not independently associated with postoperative complications, overall survival (OS) or disease-free survival (DFS), except for NRS-2002 ≥3 as an independent risk factor of OS. Sarcopenia was always an independent risk factor for postoperative complications, OS, and DFS after adjusting for nutritional risk and the other covariates in the multivariate analyses.

**Conclusions:** MUST, NRS-2002, MST, and SNAQ had low predictive power for postoperative complications and long-term survival in patients underwent radical gastrectomy for gastric cancer. Sarcopenia had additional predictive value for postoperative complications and long-term survival besides these nutritional screening tools and should be implemented in the preoperative assessments.

## Introduction

In recent years, sarcopenia has been increasingly recognized as a strong predictor for morbidity and mortality after surgery 1-3. According to the revised European consensus on definition and diagnosis of sarcopenia recommended by the European Working Group on Sarcopenia in Older People 2 (EWGSOP2), sarcopenia is diagnosed based on the measurement of muscle strength plus muscle quality/quantity 4. Computed tomography (CT) is considered to be a gold standard for the assessment of muscle mass and attenuation 4. Moreover, preoperative abdominal CT scan is necessary for patients with gastrointestinal tumors for staging of the tumor and guidance of surgery. However, muscle mass measurement before surgery is not a routine practice for radiologists in most medical centers. Therefore, sarcopenia has not been widely used in clinical practice for preoperative assessment. A recent study showed that CT measured sarcopenia may have only little additional value over Malnutrition Universal Screening Tool (MUST) for the prediction of postoperative complications after colorectal cancer surgery 5. This finding aroused us to speculate whether measurement of sarcopenia is still necessary for preoperative risk assessment in the context of nutritional risk screening before surgery for other types of malignancy, such as gastric cancer.

Malnutrition is present in about 30% of hospitalized patients, and the prevalence is especially high in patients with gastrointestinal tumors, such as gastric cancer 6, 7. There is convincing evidence that nutritional risk is associated with increased postoperative morbidity and mortality following elective surgery 8, 9. In contrast to the limited application of sarcopenia assessment in clinical practice, screening of nutritional risk has been applied in many medical centers world-widely. Therefore, it is worthwhile to investigate whether sarcopenia has additional predictive value besides nutritional screening tools for prediction of surgical prognosis when the nutritional screening has already been well implemented. Besides, although there are many nutritional screening tools, such Malnutrition Universal Screening Tool (MUST) 10, Nutrition Risk Screening 2002 (NRS-2002) 11, Malnutrition Screening Tool (MST) 12, and Short Nutritional Assessment Questionnaire (SNAQ) 13, it has as yet not been established which is the optimal nutritional assessment tool in patients scheduled to receive gastrectomy for gastric cancer.

In the present study, we aimed to investigate whether sarcopenia has additional predictive value besides 4 common nutritional screening tools (MUST, NRS-2002, MST, and SNAQ) for the prediction of postoperative complications and long-term survival after radical gastrctomy for gastric cancer.

## Methods

### Patients

From August 2014 to February 2018, patients who underwent radical gastrectomy for gastric cancer at the Gastrointestinal Surgery Department of the First Hospital Affiliated to Wenzhou Medical University were included in this prospective study. The inclusion criteria included patients who (1) were aged 18 years old or more; (2) had American Society of Anesthesiologists (ASA) grade ≤III; (3) planned to receive elective gastrectomy for gastric cancer with curative intent; (4) had preoperative abdominal CT images available for review (no more than 1 month before operation); and (5) agreed to take part in this study and signed the informed consent. Exclusion criteria included patients who (1) were aged <18 years; (2) were unable to be assessed for nutritional status due to mental disorder or disturbance of consciousness; (3) were unable be measured for muscle strength or physical performance due to physical deformity; (4) declined to take part in the study; and (5) underwent palliative operation. This project was approved by the Ethical Review Board of The First Affiliated Hospital of Wenzhou Medical University and registered in China Clinical Trial Registry (NO. ChiCTR1800019717). All patients received standard managements and treatments following the Japanese gastric cancer treatment guidelines 2010 (version 3) 14.

### Data collection

The following data were collected prospectively: (1) preoperative patient and disease characteristics, including age, gender, body mass index (BMI), plasma albumin concentration, haemoglobin concentration, ASA grade, comorbidity, tumor-node-metastasis (TNM) stage of tumor, differentiation of tumor, tumor location, previous abdominal surgery; (2) operative and treatment details, including laparoscopy-assisted surgery, type of resection, extent of lymph node dissection, combined organ resection, adjuvant chemotherapy; and (3) postoperative outcomes, including postoperative complications and mortality. Only complications classified as Grade II or above according to the Clavien-Dindo classification were analyzed in this study 15, and complications classified as Grade III or above were identified as severe complications.

### Diagnosis of sarcopenia

According to the revised European consensus on definition and diagnosis of sarcopenia recommended by EWGSOP2, sarcopenia was defined by low muscle strength plus low muscle quantity or quality. Severe sarcopenia was defined when the sarcopenic patients had additional low physical performance 4. Muscle quantity was measured using cross sectional CT images at the third lumbar vertebra (L3) level on the first image with both vertebral spines visible 16. Skeletal muscle tissues were identified by the Hounsfield units (HU) with the thresholds range of -29 to +150, using the image processing system (version 3.0.11.3 BN17 32 bit; INFINITT Healthcare Co., Ltd). Cross-sectional area of skeletal muscle tissue was normalized by the height squared to calculate the skeletal muscle index (SMI, cm^2^/m^2^). Patients with L3 skeletal muscle index <40.8 cm^2^/m^2^ for men and <34.9 cm^2^/m^2^ for women were identified as having low muscle mass 1. Muscle quality was measured by using mean muscle attenuation. The sex-specific cutoff values for defining low muscle attenuation were 28.6 HU for females and 38.5 HU for males according to our previous study 17. All patients received measurements of handgrip strength and 6-m usual gait speed tests in the first hospitalized day before surgery. Muscle strength was determined by handgrip strength tests, using an electronic hand dynamometer (EH101; CAMRY, Guangdong Province, China). Patients with handgrip strength <26 kg for men and <18 kg for women were identified as having low muscle strength. Physical performance was determined by 6-m usual gait speed tests as described previously 1, 3. Patients with 6-m usual gait speed <0.8m/s were identified as having low physical performance.

### Nutritional screening

All patients received nutritional screening using 4 common nutritional screening tools, including Malnutrition Universal Screening Tool (MUST) 10, Nutrition Risk Screening 2002 (NRS-2002) 11, Malnutrition Screening Tool (MST) 12, and Short Nutritional Assessment Questionnaire (SNAQ) 13. The MUST scores are based on BMI, unplanned weight loss, and reduced food intake for more than 5 days due to the effect of acute diseases. Patients with an MUST score of ≥1 are classified as being at nutritional risk 10. NRS-2002 identify the risk of malnutrition based on 5 variables, including general condition, BMI, weight loss, food intake over the last week, and the patients' age. Patients with an NRS 2002 score of ≥3 are classified as being at nutritional risk 11. The MST tool is based on unintentional weight loss, the extent of weight loss, and reduced food intake due to decreased appetite. Patients with an MST score of ≥2 are classified as being at nutritional risk 12. The SNAQ tool is based on unintentional weight loss, decreased appetite over the last month, and usage of supplemental drinks or tube feeding over the last month. Patients with an SNAQ score of ≥2 are classified as being at nutritional risk 13.

### Follow up

All patients were routinely followed up within the first month after surgery in the outpatient. After that, patients were followed up every 3 months for 2 years and every 6 months thereafter by telephone interviews or outpatient visits. The follow-up program was consisted of physical examination, laboratory tests, and ultrasonography and/or CT and/or endoscopy. The last follow-up date was January 2019. Overall survival (OS) was defined as the period from the time of surgery to the time of death due to any causes. Disease-free survival (DFS) was calculated from the date of surgery to the date of relapse or death due to cancer-free causes.

### Statistical analysis

Normally distributed data were presented as mean and standard deviation (SD) and compared using the t-test. Nonnormally distributed data were presented as median and interquartile range (IQR) and compared using the Mann-Whitney U-test. Categorical data were compared using the chi-square test or Fisher exact test. Univariate analysis was used to identify potential risk factors for postoperative complications, overall survival and disease-free survival. Multivariate logistic regression analysis or Cox proportional-hazards analysis included variables with a P-value of <0.10 in the univariate analysis, as well as other clinical relevant variables, including age, gender, BMI or adjuvant chemotherapy. Survival curves were estimated using the Kaplan-Meier method and compared using log-rank tests. All data were analyzed using SPSS statistics version 22.0 (IBM, USA). Two-tailed P values <0.05 were considered statistically significant.

## Results

From August 2014 to February 2018, 948 patients who underwent gastrectomy for gastric cancer met our inclusion criteria initially. Sixty-eight patients were excluded for reasons of underwent palliative surgery, unable to be assessed for muscle strength or physical performance, failed to be screened for nutritional status or refused to participate in the study. Finally, a total of 880 patients were included in the analyses.

Sarcopenia was identified in 167 (18.98%) of the patients, in which 53 (6.02%) patients were identified as having severe sarcopenia. Compared with nonsarcopenic patients, patients with sarcopenia had older age, higher ASA grade, higher TNM stage, lower BMI, lower L3 SMI, lower muscle attenuation, lower handgrip strength, lower gait speed, lower preoperative albumin and hemoglobin levels, and received less laparoscopy-assisted surgery (Table 1). The incidence of nutritional risk identified by the 4 nutritional screening tools in our patients were 44.66% (MUST ≥1), 35.23% (NRS-2002 ≥3), 29.89% (MST ≥2), and 20.34% (SNAQ ≥2). Sarcopenia showed a significant correlation with nutritional risk identified by all of 4 nutritional screening tools (Table 1). However, the agreement of sarcopenia with nutritional risk is slight (kappa <0.2).

In the 880 included patients, 231 (26.25%) occurred postoperative complications graded II and above (Table 2). The details of postoperative complications were listed in Table S1. Univariate analyses showed that MUST ≥1, NRS-2002 ≥3, SNAQ ≥2, sarcopenia, age ≥75 years, ASA=III, Charlson comorbidity index ≥1, anemia, hypoproteinemia, tumor located at cardia, and laparoscopic surgery were significantly associated with postoperative complications (Table 2). None of the 4 nutritional screening tools had good sensitivity for the prediction of postoperative complications, ranging from 26.84% to 51.08% (Table S2). Sarcopenia had a high specificity (86.29%) but low sensitivity (33.77%) for the prediction of postoperative complications (Table S2). To further compare the predictive values of the 4 nutritional screening tools on the postoperative complications, we performed 4 separate multivariate analyses, adjusting the effect of the 4 nutritional screening tools for the same covariates, including gender, age, BMI, Charlson comorbidity index, anemia, hypoproteinemia, tumor location, laparoscopic surgery, and sarcopenia. None of these nutritional screening tools remained independently associated with postoperative complications after adjusting for these covariates in the multivariate analyses. Sarcopenia, tumor located at cardia, and Charlson comorbidity index ≥1 were the independent risk factors for postoperative complications in all of the 4 multivariate analyses (Table 3).

Univariate analyses and Kaplan-Meier analyses showed that MUST ≥1, MST ≥2, SNAQ ≥2, and NRS-2002 ≥3 were all associated with worse OS (Table 4, Figure S1). However, in the multivariate analyses, only NRS-2002 ≥3 remained an independent risk factor for OS. Other independent risk factors for OS were sarcopenia, higher TNM stage, undifferentiated tumor histological type, total gastrectomy in all of the 4 multivariate analyses, whereas adjuvant chemotherapy was an independent protective factor (Table 5). Univariate analyses and Kaplan-Meier analyses showed that MUST ≥1, MST ≥2 and SNAQ ≥2 were associated with worse DFS (Table 4, Figure S1). However, none of these nutritional screening tools remained significant in the multivariate analyses for DFS. Sarcopenia, higher TNM stage, undifferentiated tumor histological type, total gastrectomy were independently associated the worse DFS in all of the 4 multivariate analyses, whereas adjuvant chemotherapy was an independent protective factor (Table 6).

## Discussion

The 4 nutritional screening tools in the present study were initially developed to screen the nutritional risk. MUST was developed by the Malnutrition Advisory Group for application to all adult patients across all health care settings. It is supported by many governmental and non-governmental organizations and is the commonly used globally, especially in the European countries 10. NRS-2002 was created on the basis of its power to predict the therapeutic effect of nutritional support 11, and was recommend by ESPEN for the screening of nutritional risk in hospitalized patients 6. MST was developed based on its predictive ability of subjective global assessment (SGA) in mixed hospital patient population 12. SNAQ was created based on its predictive values for nutritional status in patients in the mixed internal and surgery/oncology wards 13. In the present study, we compared the 4 nutritional screening tools for their predictive value for postoperative outcomes after gastrectomy for gastric cancer. We found that most of these nutritional screening tools showed significant association with postoperative complications, OS, and DFS, suggesting the close relationship between nutritional status and postoperative outcomes. However, none of these tools showed a good sensitivity for the prediction of postoperative complications, ranging from 26.84% to 51.08%. Receiver operating characteristic (ROC) curves showed a low predictive power of postoperative complications for the 4 tools, ranging from 0.532 to 0.561 (Table S2). Moreover, none of these tools were independent risk factors for postoperative complications or long-term survival in the multivariate analyses, except for NRS2002 as an independent risk factor for overall survival. This indicated that NRS-2002 had a superior predictive value for postoperative survival over the other 3 nutritional screening tools. Based on the above findings, we suggested that nutritional screening is not enough for surgical risk assessment in patients underwent radical gastrectomy for gastric cancer.

In the multivariate analyses, sarcopenia always remained an independent risk factor for postoperative complications and long-term survival after adjusting for nutritional risk determined by each of these nutritional screening tools and the other covariates. This result strongly demonstrated the additional predictive value of sarcopenia on postoperative complications and long-term survival over all of these nutritional screening tools. One of our recent study showed that sarcopenia was an independent risk factor for postoperative complications after gastrectomy in patients without nutritional risk 18, which also demonstrated the additional predictive value of sarcopenia over the nutritional screening tools.

Few studies have compared the value of sarcopenia with nutritional screening tools for the prediction of postoperative outcomes. A recent study by Kroft et al. showed sarcopenia measured by CT have only little additional value over the MUST for the prediction of postoperative morbidity after colorectal cancer surgery, which was in contrast with our findings 5. The reason of the inconsistent results can be explained by the following factors. First, in the study of Kroft et al., sarcopenia was diagnosed by measuring skeletal muscle quantity/quality only. However, the functional aspects of sarcopenia, including muscle strength and physical performance, are also significant for the prediction of postoperative outcomes 1, 3. Second, the study of Kroft et al. was based on patients underwent colorectal cancer surgery, which has certain differences with our patient underwent gastrectomy for gastric cancer. However, the present study and the previous study by Kroft et al. are all single-center studies including patients underwent surgery for homogeneous types of malignancy. It is unknown whether the predictive value of sarcopenia besides nutritional risk is dependent on certain types of surgery or universal among general major abdominal surgeries. Therefore, we suggest that future multi-center studies including patients with various types of malignancy were needed to further demonstrate the predictive value of sarcopenia besides nutritional risk for the postoperative outcomes.

Although the 4 nutritional screening tools showed a significant correlation with sarcopenia, their agreements with sarcopenia was low (kappa <0.2), because they focus on different aspects in their components of definition compared with sarcopenia. Generally, these nutritional screening tools mainly focus on the BMI, weight loss or reduced intake of food. However, none of these tools distinguish the body compositions. Different body compositions have distinct impact on the surgical outcomes. Low muscle mass has been recognized to be associated with poor clinical outcomes 19, 20. However, visceral obesity has been found to be a risk factor for postoperative complications 21, 22. Sarcopenic obesity, which is characterized by low muscle mass and high BMI or high visceral fat mass, was associated with negative clinical outcomes 23, 24. However, these patients cannot be identified by traditional nutritional screening tools. One important feature of sarcopenia is the loss of muscle mass. Therefore, measurement of sarcopenia could add significant information for risk assessment before surgery. Moreover, physical function has been increasing recognized to be associated with adverse postoperative outcomes. Reddy et al. reported that timed stair climbing is the single strongest predictor of perioperative complications in patients undergoing abdominal surgery, even better than the ACS NSQIP calculator 25. Sato et al. showed that low hand grip strength was a significant risk factor for morbidity after gastric cancer surgery 26. Physical function is another important component of sarcopenia, which can also explain the additional predictive value of sarcopenia over nutritional screening tools. In the present study, we used the revised European consensus on definition and diagnosis of sarcopenia recommended by EWGSOP2 4. Compared with the previous consensus of sarcopenia definition recommended by EWGSOP1 27, the revised consensus added low muscle quality to the diagnosis of sarcopenia. Muscle quality is a relatively new term, referring to the changes in muscle architecture and composition 28. Low muscle quality, as measured by the attenuation of the muscle has been proved to have significant influence on the prognosis after surgery 17. This can also explain the superior predictive value of sarcopenia in our study.

In conclusion, our study demonstrated that none of the 4 nutritional screening tools have strong predictive power for postoperative complications and long-term survival in patients underwent radical gastrectomy for gastric cancer. Sarcopenia had additional predictive value for postoperative complications and long-term survival besides these nutritional screening tools. Our study demonstrated the superior predictive value of sarcopenia over nutritional screening tools for the prediction of postoperative outcomes. Therefore, measurement of sarcopenia is necessary for optimizing the preoperative risk assessment for gastric cancer surgery, even when the nutritional screening has already been well implemented.

## Figures and Tables

**Table 1:** Patient demographic and clinical characteristics

Factors	All (n=880)^a^	Nonsarcopenic (n=713)^a^	Sarcopenic (n=167)^a^	*P*
Age, mean (SD), years	64.47 (10.80)	62.35 (10.35)	73.54 (7.50)	<0.001*
**Gender**				0.645
Female	230	184	46	
Male	650	529	121	
**BMI, mean (SD), kg/m^2^**	22.63 (3.07)	22.91 (3.04)	21.48 (2.96)	<0.001*
**NRS-2002 scores**				<0.001*
< 3	570	496	74	
≥ 3	310	217	93	
**MUST scores**				<0.001*
< 1	487	419	68	
≥ 1	393	294	99	
**MST scores**				0.003*
< 2	617	516	101	
≥ 2	263	197	66	
**SNAQ scores**				0.005*
< 2	701	581	120	
≥ 2	179	132	47	
**L3 SMI, mean (SD), cm^2^/m^2^**	42.73 (7.74)	43.86 (7.60)	37.89 (6.41)	<0.001*
**Muscle attenuation,mean (SD), HU**	36.12 (7.81)	37.43 (7.39)	30.54 (7.10)	<0.001*
**Handgrip strength,mean (SD), kg**	28.40 (9.22)	30.84 (8.21)	18.11 (5.25)	<0.001*
**Gait speed,median (IQR), m/s**	1.00 (0.27)	1.03 (0.25)	0.81 (0.28)	<0.001*
**Albumin, mean (SD), g/L**	38.11 (4.45)	38.71 (4.16)	35.51 (4.74)	<0.001*
**Hemoglobin,mean (SD), g/L**	120.97 (21.61)	123.52 (21.16)	110.08 (20.15)	<0.001*
**ASA grade**				<0.001*
I	208	183	25	
II	522	426	96	
III	150	104	46	
**Charlson comorbidity index**				0.088
0	622	513	109	
1	174	139	35	
≥ 2	84	61	23	
**Previous abdominal surgery**				0.145
No	777	635	142	
Yes	103	78	25	
**Tumor location**				0.579
Not at cardia	765	622	143	
At cardia	115	91	24	
**Differentiation of tumor**				0.505
Well differentiated	304	250	54	
Poorly differentiated	576	463	113	
**TNM stage**				0.001*
I	310	272	38	
II	214	167	47	
III	356	274	82	
**Extent of lymph node dissection**				0.219
D1	115	98	17	
D2	765	615	150	
**Type of resection**				0.103
Subtotal gastrectomy	549	454	95	
Total gastrectomy	331	259	72	
**Combined resection**				0.069
No	810	662	148	
Yes	70	51	19	
**Laparoscopy-assisted surgery**				0.001*
No	644	504	140	
Yes	236	209	27	

a:Values are number of patients unless indicated otherwise; *Statistically significant; SD, standard deviation; BMI, body mass index; L3 SMI, third lumbar vertebra skeletal muscle index; IQR, interquartile range; ASA, American Society of Anesthesiologists; TNM, tumor-node-metastasis.

**Table 2:** Univariate analysis for risk factors associated with postoperative complications

	Without complications (n=649)	With complications (n=231)	*P*
**MUST**			0.022*
<1	374	113	
≥1	275	118	
**NRS-2002**			0.001*
<3	441	129	
≥3	208	102	
**MST**			0.067
<2	466	151	
≥2	183	80	
**SNAQ**			0.004*
<2	532	169	
≥2	117	62	
**Sarcopenia**			<0.001*
No	560	153	
Yes	89	78	
**Gender**			0.144
Female	178	52	
Male	471	179	
**Age**			<0.001*
<75 y	555	166	
≥75 y	94	65	
**BMI**			0.285
≥18.5	604	210	
<18.5	45	21	
**ASA**			0.001*
I, II	555	175	
III	94	56	
**Charlson comorbidity index**			<0.001*
0	482	140	
≥1	167	91	
**Anemia**			<0.001*
No	439	117	
Yes	210	114	
**Hypoproteinemia**			<0.001*
No	522	155	
Yes	127	76	
**Previous abdominal surgery**			0.155
No	579	198	
Yes	70	33	
**TNM stage**			0.188
I	240	70	
II	153	61	
III	256	100	
**Tumor differentiation**			0.974
Differentiated	224	80	
Undifferentiated	425	151	
**Tumor location**			0.007*
Not cardia	576	189	
Cardia	73	42	
**Type of resection**			0.110
Subtotal gastrectomy	415	134	
Total gastrectomy	234	97	
**Laparoscopic surgery**			0.010*
No	460	184	
Yes	189	47	
**Combined organ resection**			0.305
No	601	209	
Yes	48	22	
**Extent of lymph node dissection**			0.619
D1	87	28	
D2	562	203	

*Statistically significant (*P* < 0.05); BMI, body mass index; ASA, American Society of Anesthesiologists; TNM, tumor-node-metastasis.

**Table 3:** Multivariate analysis for risk factors associated with postoperative complications

	MUST ≥1		NRS-2002 ≥3		MST ≥2		SNAQ ≥2	
	OR (95%CI)	*P*	OR (95%CI)	*P*	OR (95%CI)	*P*	OR (95%CI)	*P*
Nutritional risk	1.172 (0.834-1.647)	0.361	1.198 (0.847-1.694)	0.308	1.098 (0.775-1.555)	0.600	1.417 (0.968-2.073)	0.073
Sarcopenia	2.270 (1.523-3.383)	<0.001*	2.252 (1.509-3.360)	<0.001*	2.288 (1.536-3.410)	<0.001*	2.270 (1.522-3.385)	<0.001*
Gender (male/female)	1.247 (0.856-1.818)	0.251	1.245 (0.855-1.815)	0.253	1.246 (0.853-1.820)	0.255	1.256 (0.862-1.830)	0.236
Age ≥75 y	1.443 (0.962-2.165)	0.076	1.412 (0.940-2.123)	0.097	1.430 (0.952-2.146)	0.085	1.455 (0.968-2.187)	0.071
BMI <18.5	0.971 (0.524-1.799)	0.926	0.987 (0.539-1.807)	0.966	1.035 (0.570-1.879)	0.910	1.016 (0.559-1.847)	0.958
Charlson comorbidity index ≥1	1.838 (1.319-2.562)	<0.001*	1.823 (1.308-2.542)	<0.001*	1.821 (1.306-2.540)	<0.001*	1.819 (1.304-2.537)	<0.001*
Anemia	1.407 (0.979-2.021)	0.065	1.422 (0.991-2.039)	0.056	1.426 (0.994-2.046)	0.054*	1.399 (0.974-2.008)	0.069
Hypoproteinemia	1.243 (0.827-1.869)	0.295	1.225 (0.813-1.846)	0.333	1.251 (0.833-1.881)	0.280	1.219 (0.809-1.835)	0.344
Tumor located at cardia	1.784 (1.154-2.756)	0.009*	1.759 (1.138-2.719)	0.011*	1.777 (1.150-2.746)	0.010*	1.791 (1.158-2.769)	0.009*
Laparoscopic surgery	0.785 (0.536-1.151)	0.215	0.790 (0.538-1.159)	0.277	0.780 (0.533-1.144)	0.203	0.787 (0.537-1.154)	0.220

*Statistically significant (*P* < 0.05); OR, odds ratio; CI, confidence interval; BMI, body mass index.

**Table 4:** Univariate analysis for risk factors associated with overall survival and disease-free survival

	Overall survival		Disease-free survival	
	HR (95% CI)	*P*	HR (95% CI)	*P*
MUST ≥1	1.765 (1.333-2.336)	<0.001*	1.474 (1.097-1.982)	0.010*
NRS-2002 ≥3	1.884 (1.427-2.489)	<0.001*	1.269 (0.937-1.719)	0.124
MST ≥2	1.632 (1.226-2.173)	0.001*	1.446 (1.061-1.971)	0.020*
SNAQ ≥2	1.554 (1.135-2.129)	0.006*	1.892 (1.370-2.611)	<0.001*
Sarcopenia	2.540 (1.855-3.423)	<0.001*	2.295 (1.662-3.170)	<0.001*
Gender (male/female)	1.136 (0.819-1.576)	0.445	1.063 (0.756-1.496)	0.725
Age ≥75 y	1.744 (1.268-2.398)	0.001*	1.296 (0.898-1.870)	0.165
BMI <18.5	1.567 (0.997-2.463)	0.052	1.515 (0.930-2.467)	0.095
ASA ≥III	1.620 (1.176-2.232)	0.003*	1.661 (1.181-2.337)	0.004*
Charlson comorbidity index ≥1	1.056 (0.777-1.437)	0.726	1.240 (0.904-1.701)	0.183
Anemia	1.790 (1.355-2.363)	<0.001*	1.892 (1.408-2.543)	<0.001*
Hypoproteinemia	1.645 (1.221-2.215)	0.001	1.764 (1.290-2.413)	<0.001*
Previous abdominal surgery	0.945 (0.763-1.170)	0.602	1.207 (0.779-1.872)	0.400
**TNM stage**		<0.001*		
II/I	2.627 (1.494-4.620)	0.001*	5.802 (2.885-11.668)	<0.001*
III/I	8.502 (5.270-13.717)	<0.001*	14.994 (7.875-28.549)	<0.001*
Tumor differentiation Undifferentiated/Differentiated	2.404 (1.694-3.413)	<0.001*	2.813 (1.908-4.147)	<0.001*
Tumor location at cardia	1.191 (0.803-1.766)	0.384	1.145 (0.750-1.745)	0.531
Type of resection (Total gastrectomy/Subtotal gastrectomy	2.096 (1.586-2.776)	<0.001*	2.121 (1.577-2.853)	<0.001*
Laparoscopic surgery	0.453 (0.306-0.672)	<0.001*	0.504 (0.338-0.751)	0.001*
Combined organ resection	1.904 (1.252-2.896)	0.003*	2.176 (1.415-3.348)	<0.001*
Extent of lymph node dissection (D2/D1)	1.225 (0.805-1.865)	0.343	1.273 (0.807-2.009)	0.300
Adjuvant chemotherapy	0.813 (0.616-1.073)	0.144	1.062 (0.788-1.430)	0.693
**Postoperative complications**		0.002*		0.257
Grade II/ No	1.592 (1.146-2.210)	0.006*	1.258 (0.872-1.816)	0.220
Grade III-V/ No	1.887 (1.166-3.054)	0.010*	1.433 (0.825-2.491)	0.202

*Statistically significant (*P* < 0.05); HR, hazard Ratio; CI, confidence interval; BMI, body mass index; ASA, American Society of Anesthesiologists; TNM, tumor-node-metastasis.

**Table 5:** Multivariate analysis for risk factors associated with overall survival

	MUST ≥1		NRS-2002 ≥3		MST ≥2		SNAQ ≥2	
	HR (95%CI)	*P*	HR (95%CI)	*P*	HR (95%CI)	*P*	HR (95%CI)	*P*
Nutritional risk	1.192 (0.878-1.619)	0.260	1.391 (1.029-1.878)	0.032*	1.159 (0.859-1.564)	0.335	1.007 (0.852-1.189)	0.937
Sarcopenia	1.609 (1.141-2.268)	0.007*	1.562 (1.108-2.201)	0.011*	1.598 (1.134-2.252)	0.007*	1.605 (1.137-2.264)	0.007*
Gender (male/female)	1.189 (0.846-1.669)	0.319	1.178 (0.840-1.653)	0.343	1.183 (0.843-1.660)	0.330	1.165 (0.831-1.634)	0.377
Age ≥75 y	1.042 (0.716-1.515)	0.831	1.022 (0.704-1.484)	0.909	1.030 (0.708-1.498)	0.877	1.042 (0.717-1.514)	0.829
BMI <18.5	1.249 (0.765-2.039)	0.373	1.209 (0.748-1.954)	0.439	1.308 (0.812-2.106)	0.270	1.355 (0.843-2.178)	0.209
ASA ≥III	1.129 (0.804-1.586)	0.485	1.110 (0.789-1.562)	0.548	1.118 (0.796-1.570)	0.520	1.118 (0.796-1.569)	0.520
Anemia	1.071 (0.779-1.474)	0.672	1.079 (0.785-1.482)	0.640	1.073 (0.779-1.476)	0.668	1.083 (0.787-1.491)	0.624
Hypoproteinemia	0.882 (0.624-1.246)	0.475	0.847 (0.599-1.197)	0.346	0.904 (0.642-1.275)	0.566	0.904 (0.639-1.277)	0.566
Undifferentiated Histologic type	1.532 (1.058-2.218)	0.024*	1.528 (1.056-2.211)	0.025*	1.549 (1.070-2.240)	0.020*	1.544 (1.067-2.234)	0.021*
**TNM stage**		<0.001*		<0.001*		<0.001*		
II/I	2.623 (1.438-4.784)	0.002*	2.619 (1.437-4.776)	0.002*	2.638 (1.446-4.812)	0.002*	2.688 (1.475-4.898)	0.001*
III/I	8.059 (4.736-13.711)	<0.001	8.265 (4.865-14.040)	<0.001*	8.131 (4.783-13.821)	<0.001*	8.306 (4.889-14.114)	<0.001*
Total gastrectomy	1.569 (1.173-2.098)	0.002*	1.566 (1.171-2.094)	0.002*	1.571 (1.175-2.101)	0.002*	1.589 (1.189-2.124)	0.002*
Laparoscopic surgery	0.888 (0.588-1.342)	0.574	0.915 (0.605-1.386)	0.676	0.878 (0.582-1.326)	0.536	0.877 (0.580-1.325)	0.533
Combined resection	1.257 (0.812-1.945)	0.305	1.252 (0.809-1.936)	0.313	1.219 (0.786-1.892)	0.377	1.249 (0.808-1.932)	0.317
**Postoperative complications**		0.358		0.333		0.335		0.315
Grade II/ No	1.218 (0.863-1.719)	0.262	1.221 (0.865-1.724)	0.255	1.230 (0.872-1.735)	0.239	1.231 (0.872-1.736)	0.238
Grade III-V/No	1.349 (0.805-2.259)	0.256	1.370 (0.818-2.293)	0.231	1.352 (0.808-2.263)	0.251	1.376 (0.819-2.309)	0.228
Adjuvant chemotherapy	0.444 (0.326-0.603)	<0.001*	0.436 (0.321-0.594)	<0.001*	0.440 (0.323-0.599)	<0.001*	0.444 (0.326-0.605)	<0.001*

*Statistically significant (*P* < 0.05); HR, hazard Ratio; CI, confidence interval; BMI, body mass index; ASA, American Society of Anesthesiologists; TNM, tumor-node-metastasis.

**Table 6:** Multivariate analysis for risk factors associated with disease-free survival

	MUST ≥1		NRS-2002 ≥3		MST ≥2		SNAQ ≥2	
	HR (95%CI)	*P*	HR (95%CI)	*P*	HR (95%CI)	*P*	HR (95%CI)	*P*
Nutritional risk	0.936 (0.678-1.292)	0.687	0.853 (0.615-1.184)	0.342	1.009 (0.733-1.389)	0.956	0.893 (0.755-1.056)	0.186
Sarcopenia	1.611 (1.121-2.314)	0.011*	1.636 (1.136-2.356)	0.008*	1.609 (1.119-2.312)	0.010*	1.579 (1.097-2.274)	0.014*
Gender (male/female)	1.033 (0.726-1.470)	0.855	1.039 (0.732-1.475)	0.831	1.042 (0.733-1.482)	0.817	1.060 (0.746-1.506)	0.746
Age ≥75 y	0.732 (0.480-1.118)	0.149	0.738 (0.483-1.128)	0.160	0.731 (0.478-1.118)	0.149	0.725 (0.474-1.109)	0.139
BMI <18.5	1.524 (0.897-2.591)	0.119	1.561 (0.927-2.629)	0.094	1.474 (0.885-2.456)	0.136	1.454 (0.876-2.414)	0.148
ASA ≥III	1.351 (0.946-1.929)	0.098	1.353 (0.948-1.931)	0.096	1.356 (0.950-1.936)	0.094	1.356 (0.949-1.937)	0.094
Anemia	1.153 (0.823-1.614)	0.408	1.153 (0.823-1.614)	0.408	1.149 (0.821-1.609)	0.418	1.142 (0.815-1.599)	0.440
Hypoproteinemia	1.011 (0.701-1.458)	0.954	1.029 (0.711-1.488)	0.880	1.002 (0.697-1.441)	0.992	0.971 (0.674-1.400)	0.876
Undifferentiated Histologic type	1.727 (1.154-2.585)	0.008*	1.734 (1.159-2.594)	0.007*	1.720 (1.150-2.573)	0.008*	1.714 (1.146-2.563)	0.009*
**TNM stage**		<0.001*		<0.001*		<0.001*		<0.001*
II/I	6.180 (2.978-12.824)	<0.001*	6.199 (2.990-12.850)	<0.001*	6.122 (2.951-12.702)	<0.001*	6.063 (2.923-12.573)	<0.001*
III/I	14.374 (7.235-28.588)	<0.001*	14.271 (7.192-28.297)	<0.001*	14.215 (7.157-28.234)	<0.001*	13.915 (7.007-27.633)	<0.001*
Total gastrectomy	1.530 (1.124-2.082)	0.007*	1.528 (1.124-2.078)	0.007*	1.521 (1.118-2.070)	0.008*	1.517 (1.116-2.063)	0.008*
Laparoscopic surgery	0.958 (0.631-1.453)	0.839	0.941 (0.619-1.430)	0.775	0.961 (0.634-1.457)	0.851	0.974 (0.642-1.478)	0.902
Combined resection	1.459 (0.939-2.267)	0.093	1.458 (0.939-2.264)	0.093	1.466 (0.943-2.279)	0.089	1.489 (0.958-2.315)	0.077
**Postoperative complications**		0.952		0.952		0.957		0.992
Grade II/ No	0.983 (0.671-1.438)	0.928	0.984 (0.673-1.440)	0.935	0.978 (0.669-1.431)	0.910	0.977 (0.667-1.430)	0.905
Grade III-V/No	1.086 (0.605-1.950)	0.783	1.087 (0.606-1.950)	0.778	1.076 (0.598-1.935)	0.807	1.004 (0.551-1.826)	0.991
Adjuvant chemotherapy	1.727 (1.154-2.585)	0.008*	0.474 (0.342-0.658)	<0.001*	0.471 (0.340-0.654)	<0.001*	0.465 (0.335-0.645)	<0.001*

*Statistically significant (*P* < 0.05); HR, hazard Ratio; CI, confidence interval; BMI, body mass index; ASA, American Society of Anesthesiologists; TNM, tumor-node-metastasis.

## Abbreviations

EWGSOP: European Working Group on Sarcopenia in Older People
CT: computed tomography
MUST: Malnutrition Universal Screening Tool
NRS-2002: Nutrition Risk Screening 2002
MST: Malnutrition Screening Tool
SNAQ: Short Nutritional Assessment Questionnaire
ASA: American Society of Anesthesiologists
BMI: body mass index
TNM: tumor-node-metastasis
L3: third lumbar vertebra
HU: Hounsfield units
SMI: skeletal muscle index
OS: overall survival
DFS: disease-free survival
SD: standard deviation
IQR: interquartile range
OR: odds ratio
HR: hazard Ratio
CI: confidence interval
ROC: receiver operating characteristic
