# Supplementary material for: Value of Sarcopenia defined by the new EWGSOP2 consensus for the prediction of Postoperative Complications and Long-term Survival after Radical Gastrectomy for Gastric Cancer: A comparison with four common nutritional screening tools
Source: J Cancer. 2020 Aug 6;11(19):5852–60. doi: 10.7150/jca.49815 (PMC7477454; doi:10.7150/jca.49815)
Supplement: Supplementary file 1 — Supplementary figures and tables. [file jcav11p5852s1.pdf]

Table S1. Details of postoperative complications

	All (n = 880)	Nonsarcopenia (n=713)	Sarcopenia (n=167)[a]	P
Total postoperative complications*	231 (26.2)	153 (21.5)	78 (46.7)	<0.001*
Severe complications[#]	58 (6.6)	35 (4.9)	23 (13.8)	<0.001*
**Grade II**	173 (19.6)	118 (16.5)	55 (32.9)	<0.001*
Pneumonia	32	15	17	
Intra-abdominal infection	27	24	3	
Gastrointestinal dysfunction[&]	25	15	10	
Wound infection	14	12	2	
Small bowel obstruction	13	8	5	
Duodenal fistula	9	6	3	
Venous thrombosis	9	7	2	
Intra-abdominal hemorrhage	8	6	2	
Pulmonary atelectasis	6	3	3	
Seroperitoneum	6	5	1	
Anastomotic leakage	5	4	1	
Lymphatic fistula	4	2	2	
Pancreatic fistula	3	2	1	
Blood Infection	3	3	0	
Gastrointestinal hemorrhage	3	3	0	
Wernicke's encephalopathy	2	1	1	
Arrhythmia	1	1	1	
Pulmonary embolism	1	1	0	
Urinary system infection	1	0	1	
Intestinal infection	1	0	0	
**Grade III**	31 (3.5)	24 (3.4)	7 (4.2)	0.602
Intra-abdominal hemorrhage	15	14	1	
Anastomotic leakage	7	4	3	
Pleural effusion	3	1	2	
Intra-abdominal infection	3	2	1	
Biliary fistula	1	1	0	
Pancreatic fistula	2	2	0	
**Grade IV**	23 (2.6)	10 (1.4)	13 (7.8)	<0.001*
Heart failure	5	3	2	
Anastomotic leakage	1	1	0	
Anastomotic stenosis	1	0	1	
Cerebral infarction	1	1	0	
Intra-abdominal hemorrhage	1	0	1	
Multiple organ failure	2	0	2	
Pancreatic fistula	1	1	0	
Pneumonia	1	0	1	
Pulmonary embolism	1	0	1	
Respiratory failure	5	2	3	

Septic shock	4	1	3	
**Grade V**	4 (0.5)	1 (0.1)	3 (1.8)	0.026*
Intra-abdominal hemorrhage	3	1	2	
Septic shock	1	0	1	

*Complications classified as grade II and above.

Complications classified as grade III and above.

[&] Complications requiring total parenteral nutrition support, including delayed gastric emptying, prolonged postoperative ileus, etc.

The number in the table indicates the number of patients and percent.

Table S2. Sensitivity and specificity of nutritional tools and sarcopenia in the prediction of postoperative complications

Factors	Sensitivity%	Specificity%	ROC (95% CI)
MUST ≥1	51.08	57.63	0.544 (0.500-0.587)
NRS-2002 ≥3	44.16	67.95	0.561 (0.517-0.604)
MST ≥2	34.63	70.80	0.532 (0.488-0.576)
SNAQ ≥2	26.84	81.97	0.544 (0.500-0.588)
Sarcopenia	33.77	86.29	0.600 (0.556-0.645)

ROC, Receiver operating characteristic; CI, confidence interval.

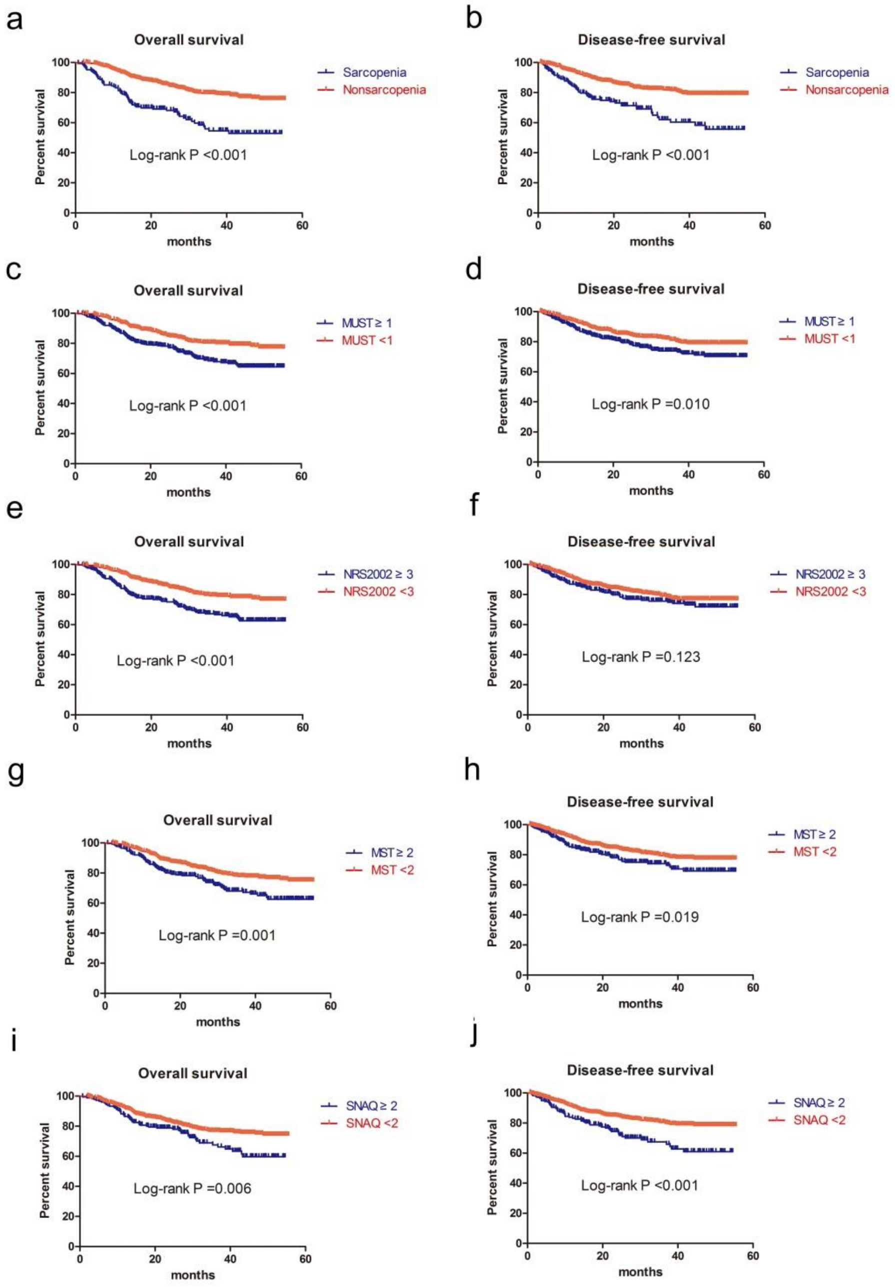

Figure S1. Kaplan Meier curves for overall survival and disease-free survival in patients with/without sarcopenia and nutritional risk (**a, b** sarcopenia vs nonsarcopenia; **c, d** MUST ≥1 vs MUST <1; **e, f** NRS-2002 ≥3 vs NRS-2002 <3; **g, h** MST ≥2 vs MST <2; **i, j** SNAQ ≥2 vs SNAQ <2).
